# Synergistic effect of ursolic acid and piperine in CCl_4_ induced hepatotoxicity

**DOI:** 10.1080/07853890.2021.1995625

**Published:** 2021-11-09

**Authors:** Sayan Biswas, Amit Kar, Nanaocha Sharma, Pallab K. Haldar, Pulok K. Mukherjee

**Affiliations:** a School of Natural Product Studies, Department of Pharmaceutical Technology, Jadavpur University, Kolkata, India; b Department of Biotechnology, Institute of Bioresources and Sustainable Development, Imphal, India

**Keywords:** Hepatoprotective, isobologram, combination index, pharmacokinetics

## Abstract

**Background:**

Ursolic acid (UA) is a potent plant-based hepatoprotective agent having poor bioavailability, which hampers its therapeutic efficacy. The present study tries to overcome this limitation by combining it with piperine (PIP), a proven bioenhancer and hepatoprotective agent.

**Methods:**

The type of interaction (synergism, addition, or antagonism) resulting between UA and PIP was analyzed and quantified by isobologram and combination index analysis. The hepatoprotective activity of UA and PIP was evaluated by measuring the level of hepatic marker enzymes. Pharmacokinetic analysis was carried out to ascertain the improvement of bioavailability.

**Results:**

The combinations significantly decrease the enzyme levels, which indicate better hepatoprotective activity compared to single drugs. The relative oral bioavailability of UA was increased about tenfold (from AUC_0–_*_t_* =12.78 ± 2.59 µg/h/ml to 125.15 ± 1.84 µg/h/ml) along with the improvement of plasma concentration and elimination half-life.

**Conclusions:**

The findings indicated that the combination of PIP and UA is an effective strategy in enhancing the bioavailability and hepatoprotective potential of UA.

**KEY MESSAGES**
- Ursolic acid in a combination with piperine provides a synergistic hepatoprotective effect in carbon tetrachloride induced liver damage in rats.
- Piperine improves the pharmacokinetic properties of ursolic acid when given in combination.
- Piperine improves the relative oral bioavailability of ursolic acid by tenfold when combined together.

## Introduction

Many of the potent phytomedicines available in the market as whole extracts of plants and practitioners have always relied on the synergistic interactions between the components of individual or mixtures of herbs of these formulations to play a vital part in their therapeutic efficacy. The mechanism of action of many herbal formulations containing herbal extracts is still unknown and there are several reports observed where a total herb extract was found to provide a better effect than an equivalent dose of an isolated compound. One of the most important examples of such formulation is available in the Indian traditional medicinal system “Ayurveda” which uses many fixed combination formulae with “Trikatu” featuring in many of them. This mixture contains black pepper (*Piper nigrum*), long pepper (*Piper longum*) and ginger (*Zingiber officinalis*). Traditionally, this formulation is used as first-line treatment for cough, cold, fever, asthma and other respiratory problems. In a recent study, it has been shown to act by synergistic activity [1]. Similarly, another well-known ayurvedic formulation “Triphala”, a mixture of three dried fruits amla (*Emblica officinalis)*, behera (*Terminalia belerica)* and haritaki (*Terminalia chebula)* in equal proportions (1:1:1) is considered to act synergistically [2]. Drug combinations are therefore an effective treatment approach involving many pathways and targets [3,4]. Synergistic interactions on the basis of effect can be broadly classified into two categories: pharmacokinetic and pharmacodynamics. Pharmacokinetic synergism involvers improving the systemic bioavailability of drugs with a concomitant administration of another drug. The drug used to enhance the bioavailability and pharmacokinetic properties of the other drug are bio-enhancer or bio-potentiator [5]. An example of pharmacodynamics synergism is observed in Arjuna (*Terminalia arjuna*), where different saponin glycosides present in it act synergistically to improve heart muscle function resulting in enhanced pumping activity. Another such example is lemongrass (*Cymbopogon citratus*) whose essential oil contains geranial, neral, and myrcene which are antimicrobial in nature. It has been observed that the combination is more effective than individual phytomolecules against microbial growth [6].

The liver is a major body organ performing a variety of physiological and biochemical processes. Its main functions include metabolism, storage, and secretion. It is involved in detoxifying the body from endogenous waste metabolites as well as exogenous xenobiotics. The liver metabolizes carbohydrates and fats, secretes bile and stores certain vitamins. Therefore, diseases affecting normal liver functioning are a major threat to public health. Hepatic or liver disease is a collective term and encompasses cell, tissue or structural damage of liver due to microorganisms such as bacteria, virus, parasites, autoimmune disorders including immune hepatitis, primary biliary cirrhosis, etc excess drug toxicity involving drugs such as paracetamol, certain antitubercular drugs, etc), hepatotoxic compounds such as carbon tetrachloride (CCl_4_), thioacetamide, D-galactosamine/lipopolysaccharide, etc) and alcohol. Out of these compounds, Carbon tetrachloride (CCl_4_) has been extensively used to induce liver damage in animal models due to its similarity with hepatic injury in humans [7]. Carbon tetrachloride (CCl_4_) exerts its hepatotoxic action due to the formation of trichloromethyl-free radicals (–CCl_3_ or CCl_3_OO–) by cytochrome P450 enzymes (CYP 450) present in the liver. These free radicals induce lipid peroxidation in liver cells thus decreasing the antioxidant enzymes used to counter this oxidative damage. This causes damage to hepatic parenchyma cells leading to hepatotoxicity [8].

Though significant advances have been made in modern medicine, still there is a lack of effective drugs that can improve liver function, protect the liver against damage and help in the regeneration of normal liver cells [9]. The efficacy of the limited number of synthetic drugs that are available for liver treatment is controversial and presents serious side effects [10]. Major side effects of these synthetic drugs involve inflammation and cancer on long-term use. An example is tiopronin which increases the risk of liver injury by ten-fold when used for a long time. Ribavirin and interferon-α (IFN-α) combination used to treat hepatitis C patients are also detrimental to liver health on long-term use. Nexavar, a synthetic drug used to treat liver carcinoma poses side effects including diarrhoea, patchy hair loss, loss of appetite, stomach pain, etc. Sorafenib another synthetic drug used to treat advanced hepatocellular cancer may even induce renal and pancreatic failure on long-term use [11]. Therefore, there is an urgent need for a safe and effective treatment option.

Ursolic acid (UA) is a secondary plant metabolite classified as pentacyclic triterpene. It is found in the leaves, flowers and fruits of a variety of herbs [12]. Various therapeutic activities including hepatoprotective, antimicrobial, anti-inflammatory, anticancer, etc have been reported for UA [13]. However, the therapeutic potential of UA is greatly limited by its low solubility and poor membrane permeation. It is therefore classified as a class IV drug in the Biopharmaceutics Classification System having low oral bioavailability. Various drug delivery technologies have been developed for enhancing the biopharmaceutical attributes of UA. This includes nanoemulsions, mesoporous silica nanoparticles, solid lipid nanoparticles, liposomes, niossomal gels, solid dispersions, etc. [14]. Piperine (PIP) is a nitrogenous alkaloid found mainly in black pepper (*Piper nigrum* L.) and other piper species [15]. Several therapeutic activities have been attributed to PIP including anticancer [16], anti-inflammatory [17], hepatoprotective [18], antidepressant [19], neuroprotective [20], etc. Besides these therapeutic activities, PIP has been reported for increasing the bioavailability of drugs administered with it simultaneously. This is due to the inhibition potential of PIP on human CYP3A4 and P-glycoprotein (P-gp). These two are involved in the metabolism CYP3A4 and efflux (P-gp) of most of the drugs administered in the body. By inhibiting the process of metabolism and efflux, PIP enhances the bioavailability of drugs administered along with it. This is observed in various studies where the bioavailability of different classes of drugs such as non-steroidal anti-inflammatory drugs, antidiabetics, antihistamines, anti-epiletics, antiretrovirals, etc have been enhanced on simultaneous administration of PIP [15]. UA is metabolized mainly by CYP3A4 and is probably the substrate of P-gp that may be involved in the active efflux transport of UA [21]. Therefore, the present study was designed to assess the possible synergistic interaction between UA and PIP when administered simultaneously and its effect on hepatoprotection. Though various studies are reported where one phytomolecule enhances the therapeutic effect of the others such as PIP enhancing the therapeutic effect of curcumin for neuroprotection [22], hepatocellular carcinoma [23], etc. none of these studies used scientific methods to quantify the synergy observed. To the best of our knowledge, this is the first study to quantify the synergy level between two phytomolecules using isobologram and combination index analysis in animal models.

## Materials and methods

### Chemicals

UA (90.5% w/w purity) was procured from TCI, Tokyo, Japan. PIP (98% w/w purity), α-L-alanine, L-aspartate, Serum glutamic oxaloacetic transaminase (SGOT), Serum glutamic pyruvic transaminase (SGPT), and Alkaline phosphatase (ALP) were purchased from Sigma Chemical Co. (St Louis, MO). Sodium bicarbonate, potassium ferricyanide, sodium hydroxide, diethyl ether, carbon tetrachloride, olive oil and 4-aminoantipyrine were purchased from SD chemicals (Thane, India). 2, 4 dinitrophenylhydrazine and phosphate buffer saline (pH 7.4) were purchased from SRL Chemicals (Mumbai, India). High-Performance Liquid Chromatography grade (HPLC) methanol, acetonitrile and orthophosphoric acid were procured from Merck (Mumbai, India). Deionized water from Milli-Q water purification system was used for the Reverse Phase-HPLC (RP-HPLC) study.

### Experimental animals

Male Wistar rats were used for the study. They were 2–3 months of age and weighed 180–220 g. The study was carried out according to the protocol (Approval number: AEC/PHARM/1702/18/2017) approved by the animal ethical committee of the Department of Pharmaceutical Technology, Jadavpur University, India. Committee for the Purpose of Control and Supervision of Experiments on Animals (CPCSEA), Government of India, guidelines were followed to carry out the study.

### Dose selection

From the literature review, the dose of UA providing significant protection against CCl_4_ induced hepatotoxicity has been found to be 50 mg/kg p.o [24,25]. Therefore, other doses of UA were selected by taking 1/4^th^ of this dose (12.5 mg/kg), half of this dose (25 mg/kg), twice of this dose (100 mg/kg) and four times of this dose (200 mg/kg) for analysing the dose-response characteristics. Therefore, different doses selected for UA were 12.5 mg/kg, 25 mg/kg, 50 mg/kg, 100 mg/kg and 200 mg/kg. In the case of PIP from reports, it was observed that the dose at 20 mg/kg enhanced the bioavailability of compounds administered with it [26,27]. Moreover, this dose has been reported to have significant hepatoprotective activity [28]. Therefore, different doses for PIP selected were 5 mg/kg, 10 mg/kg, 20 mg/kg, 40 mg/kg and 80 mg/kg as mentioned previously in the case of UA for generating the dose-response curve.

### Experimental procedure

The rats were divided into seventeen groups each containing six (*n* = 6) animals. Animals of the normal group and CCl_4_ control group received only distilled water with Tween 20 (1% v/v) p.o. for 7 days via gastric intubation. UA12.5, UA25, UA50, UA100, UA200 groups were treated with pure UA suspension in distilled water with Tween 20 (1% v/v) at a dose level of 12.5, 25, 50, 100, and 200 mg/kg p.o., respectively for 7 days. PIP5, PIP10, PIP20, PIP40, and PIP80 groups were treated with pure PIP suspension in distilled water with Tween 20 (1% v/v) at a dose level of 5, 10, 20, 40, and 80 mg/kg p.o. respectively for 7 days. UA + PIP-1, UA + PIP-2, UA + PIP-3, UA + PIP-4, UA + PIP-5 were treated with the combination of UA and PIP at dose levels of (12.5 + 5), (25 + 10), (50 + 20), (100 + 40) and (200 + 80) mg/kg p.o respectively for 7 days.

Summarized table describing the details of the different groups (*n* = 6) 

**Table:** 

Groups	Treatment
Normal	Distilled water with Tween 20 (1% v/v) p.o. for 7 days
CCl_4_ control	Distilled water with Tween 20 (1% v/v) p.o. for 7 days
UA12.5	12.5 mg/kg of UA p.o. for 7 days
UA25	25 mg/kg of UA p.o. for 7 days
UA50	50 mg/kg of UA p.o. for 7 days
UA100	100 mg/kg of UA p.o. for 7 days
UA200	200 mg/kg of UA p.o. for 7 days
PIP5	5 mg/kg of PIP p.o. for 7 days
PIP10	10 mg/kg of PIP p.o. for 7 days
PIP20	20 mg/kg of PIP p.o. for 7 days
PIP40	40 mg/kg of PIP p.o. for 7 days
PIP80	80 mg/kg of PIP p.o. for 7 days
UA + PIP-1	12.5 mg/kg of UA p.o. and 5 mg/kg of PIP p.o. for 7 days
UA + PIP-2	25 mg/kg of UA p.o. and 10 mg/kg of PIP p.o. for 7 days
UA + PIP-3	50 mg/kg of UA p.o. and 20 mg/kg of PIP p.o. for 7 days
UA + PIP-4	100 mg/kg of UA p.o. and 40 mg/kg of PIP p.o. for 7 days
UA + PIP-5	200 mg/kg of UA p.o. and 80 mg/kg of PIP p.o. for 7 days

On the 7th day, a single dose of an equal mixture of CCl_4_ and olive oil (50% v/v, 1 ml/kg i.p.) was administered to all animals except the control group. On the 8th day, after exactly 24 h of CCl_4_ injection, all the animals were sacrificed by cervical dislocation under anaesthesia. The blood was aspirated from the left ventricle, allowed to clot in micro-centrifuge tubes at room temperature and centrifuged to collect the serum. The serum was analyzed for various markers of liver injury including serum glutamate oxaloacetate transaminase (SGOT), serum glutamate pyruvate transaminase (SGPT) and serum alkaline phosphatase (ALP) following reported methods [29].

The percent hepatoprotective activity of the samples was calculated using the following formula:

(1) (%) protection = [(a−b)/(a−c)] × 100………

where *a* is the mean value of the marker enzyme level produced by CCl_4_; *b* is the mean value of the marker enzyme level produced by CCl_4_ plus test sample, and *c* is the mean value of marker enzyme level produced by the vehicle control [30].

### Interaction analysis by isobologram method

Isobologram method and the median effect method proposed by Chou and Talalay were used to analyze the interaction between combinations of UA and PIP [31]. In this method, the different dose combinations of UA + PIP were plotted against their respective effects (ED_50_, ED_75_ and ED_90_) in the form of percent hepatoprotective effect as mentioned in Equation 1. Here ED_50_ stands for median effective dose whereby the desired therapeutic effect produced is 50% [32]. Similarly, ED_75_ and ED_90_ stand for the therapeutic effect of 75% and 90% respectively. The doses are then connected through the line of additivity. A combination was taken as synergistic, antagonistic or additive when the observed dose combination falls below, above or on the line of additivity respectively. An extended combinational effect (synergism, antagonism, additivity) for UA and PIP was also determined by median effect analysis of Chou Talalay using COMPUSYN software 2.0 to obtain a combination index, a quantitative measure of synergism. The combination index (CI) values of less than 0.3 indicate strong synergism, the value of 0.3–0.69 indicates synergism, 0.70–0.84 indicates moderate synergism, 0.85–0.89 indicates mild synergism, 0.9–1.09 indicates additive effect, 1.10–1.19 indicates slight antagonism, 1.20–1.44 indicates antagonism, >1.45 indicates moderate strong antagonism [33].

### Pharmacokinetic interaction

Male Wistar rats were divided into two main groups each containing six (*n* = 6). The first group was administered a single dose of UA (50 mg/kg) in distilled water with Tween 20 (1% v/v) p.o. via gastric intubation and the second group, UA + PIP was administered the combination (50 mg/kg +20 mg/kg) in distilled water with Tween 20 (1% v/v) p.o. via gastric intubation. Blood samples (0.5 ml) were collected from the retro-orbital plexus of rats at specific time intervals into micro-centrifuge tubes containing anticoagulant mixture. 2 ml of 0.9% v/v i.g. normal saline solution was administered to compensate rat’s body fluid after every 1.5 h. Different blood samples were centrifuged at 800xg for 10 min and plasma was separated and kept at −20 °C prior to analysis.

### RP-HPLC analysis of UA content in rat plasma

A Waters RP-HPLC system (Milford, MA) equipped with 600 quaternary pumps, Rheodyne-7725i injector with a loop size of 20 µl, a UV-Visible detector, and a C_18_ column (Waters Spherisorb, Dublin, Ireland) with a length 250 mm and width 4.6 mm having 5 µm particle size was used as stationary phase. A validated RP-HPLC method was used to analyze UA content in rat plasma with appropriate modification [34]. The mobile phase used was acetonitrile/0.5% aqueous phosphoric acid (75:25, v/v) and detection was carried out at 210 nm. UA was extracted from plasma by adding diethyl ether through vigorous vortex mixing for 3 min. The two phases were separated by centrifugation at 2500 × *g* for 7 min. The supernatant diethyl ether layer was separated and completely evaporated at 40 °C. The dry residue was reconstituted in 100 µl of mobile phase, vortex mixed for 30 s, and filtered through a 0.22 µm syringe filter prior to injecting into RP-HPLC. The flow rate was kept constant at 1 ml/min. Peaks were monitored at 210 nm.

The pharmacokinetic parameters of UA (50 mg/kg) and UA + PIP (50 mg/kg + 20 mg/kg) were obtained with the help of a computer-designed program Phoenix WinNonlin^®^ 6.4 (Certara). Maximum concentration (*C*_max_) and time to reach maximum concentration (*T*_max_) are values obtained directly from the concentration–time curve. Area under the concentration–time curve (AUC_0–_*_t_* and AUC_0–∞_), elimination half-life (*t*_1/2el_), elimination rate constant (*K*_el_), clearance (cl), the volume of distribution (*V_d_*), and mean residence time (MRT) were determined. Relative bioavailability (*F*) was calculated at a ratio of the plasma AUC (AUC_0–∞_) of the pure UA and its combination with PIP (UA + PIP).

### Statistical analysis

Data generated were analyzed statistically by one-way analysis of variance (ANOVA) followed by Tukey’s multiple comparison test using the software GraphPad Prism 5 (San Diego, CA, USA). Results were reported as mean ± standard deviation (SD) except biochemical marker enzymes study for in-vivo hepatoprotection where the results were reported as mean ± standard error of the mean (SEM). *p*-values of <.05 were considered to be statistically significant.

## Results

### Effect on SGOT, SGPT and ALP

An elevation of antioxidant enzymes SGOT, SGPT and ALP indicates loss of functional integrity of hepatic cell membranes causing leakage of these enzymes in bloodstream [7]. CCl_4_ control group exhibited a significant rise in SGOT, SGPT and ALP due to the extensive liver damage when compared to vehicle control (normal). UA12.5, UA25, UA50, UA100 and UA200 groups showed a dose-dependent decrease in marker enzyme levels as observed from Table 1. The decrease level was significant when compared to CCl_4_ control. This correlated well with the hepatoprotective property of UA as mentioned in various reports discussed previously. Similarly, PIP5, PIP10, PIP20, PIP40 and PIP80 groups also demonstrated a dose-dependent decrease in SGOT, SGPT and ALP when compared to CCl_4_ control. When combined together the marker enzyme levels decrease were highly significant (***p < .0001) for UA + PIP-1, UA + PIP-2, UA + PIP-3, UA + PIP-4 and UA + PIP-5 when compared to CCl_4_ control (Table 1) indicating the usefulness of the combination in treating hepatic damage.

**Table 1.** Effect of various doses of UA, PIP and UA + PIP on liver markers enzyme.

Group	SGOT (IU/l)	SGPT (IU/l)	ALP (IU/l)
Control	73.74 ± 1.16	59.32 ± 1.4	122.32 ± 3.4
CCl_4_ treated	130.32 ± 5.6**	100.35 ± 4.7**	220.5 ± 7.7**
UA			
12.5	115.60 ± 2.14* (26.01)	90.51 ± 1.14* (23.98)	190.06 ± 3.15* (31.04)
25	110.51 ± 2.03** (35.01)	86.39 ± 1.64** (34.02)	179.26 ± 2.17** (42.04)
50	97.50 ± 2.03 ** (58.06)	77.37 ± 1.34** (56.07)	160.61 ± 1.27** (61.02)
100	92.97 ± 1.63** (66.01)	72.86 ± 1.24** (66.99)	149.81 ± 1.45** (72.04)
200	87.88 ± 1.21** (75.08)	68.35 ± 1.55** (77.99)	137.05 ± 1.25** (84.99)
PIP			
5	117.87 ± 2.24* (22.04)	89.68 ± 1.25* (26.05)	189.08 ± 1.15* (32.02)
10	105.99 ± 2.04** (43.01)	85.58 ± 1.15** (35.99)	176.32 ± 1.05** (44.99)
20	94.11 ± 1.45** (63.99)	78.60 ± 1.04** (53.09)	159.63 ± 0.76** (61.99)
40	87.88 ± 1.35** (75.08)	70.39 ± 1.74** (73.02)	146.86 ± 1.42** (75.05)
80	80.53 ± 2.15** (87.99)	66.29 ± 1.24** (83.01)	134.10 ± 2.43** (88.01)
UA + PIP			
12.5 + 5	92.98 ± 2.45*** (65.99)	69.95 ± 1.25*** (74.09)	144.90 ± 2.13*** (77.01)
25 + 10	81.09 ± 1.75*** (87.09)	65.47 ± 1.15*** (85.01)	139.99 ± 2.35*** (82.02)
50 + 20	82.79 ± 2.75*** (84.04)	64.24 ± 1.24*** (88.08)	130.17 ± 2.58*** (92.04)
100 + 40	87.32 ± 2.55*** (75.99)	61.37 ± 1.14*** (95.03)	128.21 ± 2.18*** (94.08)
200 + 80	76.57 ± 2.05*** (94.99)	59.73 ± 1.34*** (99.07)	124.28 ± 2.85*** (98.03)

SGOT: serum glutamate oxaloacetate transaminase; SGPT: serum glutamate pyruvate transaminase; ALP: serum alkaline phosphatase.

Values are represented as mean ± standard error of mean (SEM); *n* = 6.

*p < .05;**p < .01; ***p < .0001. value in parenthesis indicate percent hepatoprotectivity (% hepatoprotectivity).

### Interaction between UA and PIP

**Figure 1.**
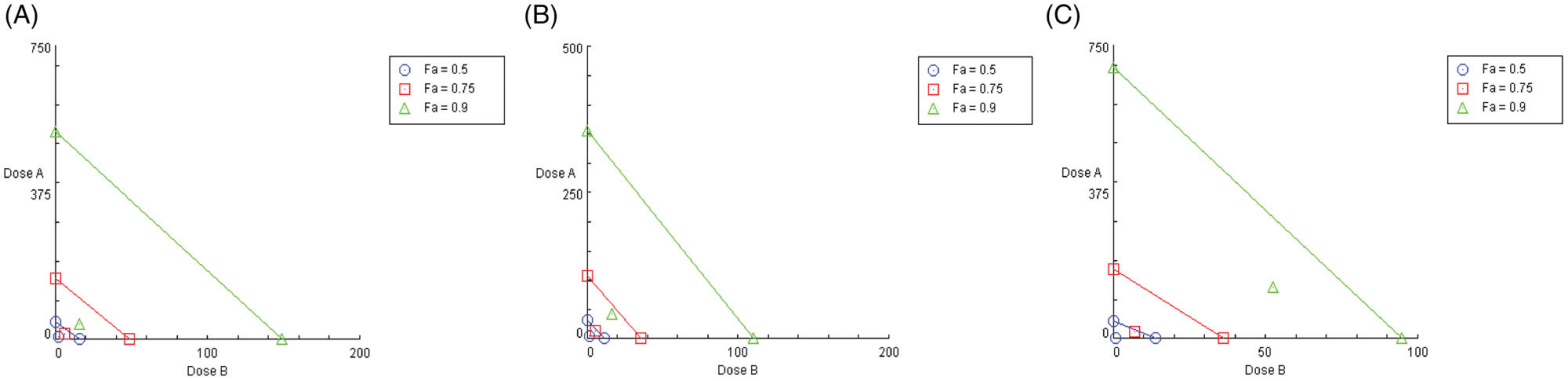
Isobologram depicting the effect of combinations (UA + PIP) on SGOT (A), SGPT (B) and ALP (C) at 50% (*F_a_* = 0.5), 75% (*F_a_* = 0.75) and 90% (*F_a_* = 0.9) effect levels. The line indicates alignment of theoretical value of an additive interaction between dose A (UA) and dose B (PIP). Values above the diagonal line of additive effects in the isobole suggest antagonism and below line suggest synergism.

The interaction of UA and PIP at all the combined doses tested exhibited significant hepatoprotective activity as observed in Table 1 when compared to the CCl_4_ control group. The type of interaction (synergism, antagonism or additivity) has been presented graphically in Figure 1. From the isobologram (Figure 1(A–C)) it is observed that the experimental data points lie at the left side of the line of additivity at all the effect levels (ED_50_, ED_75,_ ED_90_, and ED_95_) for all the marker enzymes selected. The effect levels are the effective dose of the combination showing therapeutic effects of 50%, 75%, 90% and 95% in terms of hepatoprotective activity. The combination index (CI) measures the degree of interaction between two molecules quantitatively at a given endpoint of effect. A CI value of less than 1 indicates synergism of the combination, a CI value of 1 indicates the additive effect of the combination, and a CI value greater than 1 indicates antagonism [31]. From the median effect method analysis, the value of CI obtained is shown in Table 2. All the combinations showed dose-dependent strong synergism with a CI value of less than 0.3 for SGOT, SGPT, and ALP.

**Table 2.** Combination index (CI) at the affected fractions of 50% (ED_50_), 75% (ED_70_), 90% (ED_90_) and 95% (ED_95_) of PIP combined with UA.

Liver markers	CI values ± SE			
	ED_50_	ED_75_	ED_90_	ED_95_
SGOT	0.29 ± 0.034	0.27 ± 0.012	0.18 ± 0.006	0.12 ± 0.004
SGPT	0.29 ± 0.025	0.23 ± 0.018	0.18 ± 0.015	0.15 ± 0.005
ALP	0.27 ± 0.016	0.25 ± 0.020	0.19 ± 0.017	0.14 ± 0.006

### Effect of combination on pharmacokinetic parameters

**Figure 2.**
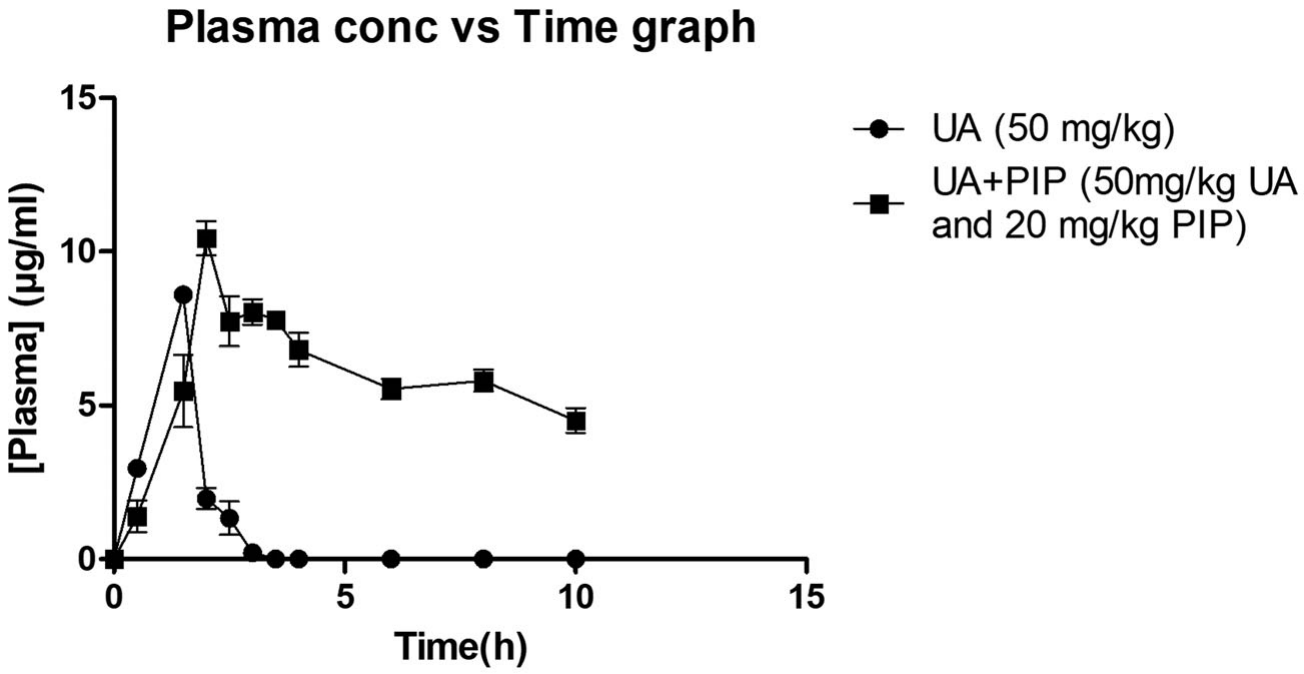
*In vivo* plasma concentration versus time profile graph for UA50 and UA + PIP (50 + 20) combination.

When combined with PIP, UA presented a significant increase in *C*_max_ and AUC_0–_*_t_* value as observed in Table 3 and Figure 2. The elimination half-life (*T*_1/2ke_) h of UA increased significantly when combined with PIP when compared to UA50 alone. A significant decrease in volume of distribution (*V_d_*), clearance (cl), and elimination rate constant (*K*_el_) were observed for the combination compared to UA alone indicating high residence time of UA in the body when combined with PIP. From the area under the curve (AUC_0–_*_t_*) indicating the relative concentration of UA with time, the relative oral bioavailability of UA in combination with PIP was found to increase by 9.79-fold compared to UA alone treated group.

**Table 3.** Pharmacokinetic profiles of pure UA50 and UA + PIP (50 + 20) combination.

Parameters	UA50	UA + PIP (50 + 20)
*C*_max_ (µg/ml)	8.4 ± 0.53	10 ± 0.56*
*T*_max_ (h)	1.7 ± 1.32	2.4 ± 1.67
*K*_el_ (1/h)	0.997 ± 0.23	0.076 ± 1.43**
*T*_1/2ke_ (h)	0.63 ± 1.54	9.14 ± 1.85**
AUC_0–_*_t_* (µg/h/ml)	12.78 ± 2.59	125.15 ± 1.84**
cl (µl/h/mg)	0.65 ± 1.77	0.074 ± 0.35**
*V_d_* (µl/g)	1.645 ± 1.12	0.95 ± 1.84**

Values are mean ± standard deviation (SD).

*p < .05; **p < .01 (significant with respect to UA 50 treated group).

## Discussion

The present study was carried out to analyze the interaction of UA when combined with PIP in alleviating CCl_4_ induced liver damage in rat models. The pharmacokinetic interaction of UA with PIP was also investigated. The hepatotoxic effect of CCl_4_ is mediated by the generation of free radicals such as trichloromethyl radical (CCl_3_) and trichloromethyl peroxy radical (CCl_3_OO) which damages the cell membrane of normal liver cells termed hepatocytes. This causes damage to hepatocytes and releases SGOT, SGPT and ALP enzymes in the systemic circulation. These enzymes are therefore useful indicators of the rate and extent of liver damage. A rise of these enzymes levels in serum is directly correlated with hepatotoxicity [34]. From the results, it is clearly observed that the combination provided significant improvement in hepatoprotective activity when compared to a single UA. This is demonstrated from the statistically significant decrease of these marker enzyme levels when compared to CCl_4_ control. In order to further inspect the type of interaction present isobologram was constructed and a combination index analysis was carried out. Isobologram is a graphical representation of various doses of two drugs at a fixed ratio [31]. From the isobologram CI can be obtained which provides a quantitative measure of the type of interaction (synergism, antagonism or addition) present in drug combination [3]. Various studies are reported whereby synergy assessment was carried out by obtaining isobologram [35]. The isobologram in the present study indicated synergism in all the combinations tested. The value of CI suggested the presence of a strong synergism between UA and PIP.

PIP has been well reported for enhancing the bioavailability of synthetic drugs as well as bioactive phytomolecules. Bi et al., (2019) have used PIP to enhance the oral bioavailability of silybin, a hepatoprotective flavonoid. When evaluated in CCl_4_ induced toxicity model the hepatoprotective effect of silybin was found to be significantly enhanced [36]. A study demonstrated significant improvement in the immunomodulatory potential of Ginsenoside Rh2 when combined with PIP. This was attributed to the increased bioavailability of Ginsenoside Rh2 when combined with PIP [37]. Similarly, Bhutani et al. [38] reported a significant increase in the antidepressant property of curcumin when combined with PIP [38]. UA on the other hand has been combined with resveratrol for promoting its anticancer activity [39]. However low solubility, coupled with poor absorption, rapid metabolism by CYP3A4 in liver microsome, and excretion by p-gp efflux transporter have limited the therapeutic potential of UA. Though the various delivery system has been developed for increasing the oral bioavailability and therapeutic efficacy of UA such as nanoparticle [40], liposome [41], nanoemulsion [42], nanosuspenison [43], etc. they are mainly concerned with increasing the solubility and permeability of UA for increasing its bioavailability [21]. This study for the first time reports the increase of oral bioavailability of UA through combination with a herbal bio enhancer. The improvement in oral bioavailability and hepatoprotective potential of UA is probably related to CYP3A4 and p-gp inhibition by PIP although further studies are required to know the exact mechanism of action.

## Conclusion

In this study, UA was combined with PIP to enhance the hepatoprotective potential of UA and to study the pharmacodynamic and pharmacokinetic interaction present in the combination. For pharmacodynamic study different doses for the combination were selected based on reports available showing the hepatoprotective activity of UA and PIP. The effect of the combinations or dose responses was represented as percent hepatoprotective activity in terms of liver marker enzymes namely SGOT, SGPT and ALP in CCl_4_ induced hepatotoxicity in a rat model. Statistical analysis of dose-response was carried out using COMPUSYN software package which uses the median effect method proposed by Chou and Talalay. Isobologram was plotted for each liver enzyme and the combination index was calculated. The study indicated the presence of strong synergism between UA and PIP in protection against CCl_4_ induced liver injury as indicated by isobologram and combination index analysis. Besides, combining UA with PIP also led to significant improvement in the pharmacokinetic properties of UA. This is indicated by an almost tenfold enhancement of relative oral bioavailability of UA when combined with PIP. Therefore, a fixed-dose combination of UA and PIP may prove to be an effective alternative to synthetic hepatoprotective agents. Further studies are required to explore this combination in different conventional drug delivery systems such as capsules, tablets, etc for commercial use.

## Data Availability

The authors confirm that the data supporting the findings of this study are available within the article.
